# Gastric Tissue Damage Analysis Generated by Ischemia: Bioimpedance, Confocal Endomicroscopy, and Light Microscopy

**DOI:** 10.1155/2013/824682

**Published:** 2013-06-15

**Authors:** Nohra E. Beltran, Laura E. Garcia, Mario Garcia-Lorenzana

**Affiliations:** ^1^Departamento de Procesos y Tecnologia, Universidad Autonoma Metropolitana, Cuajimalpa. Artificios 40, Colonia Hidalgo, 01120 Ciudad de Mexico, DF, Mexico; ^2^Centro Nacional de Investigación en Instrumentacion e Imagenologia Medica, Universidad Autonoma Metropolitana, Iztapalapa. Avenida San Rafael Atlixco 186, Colonia Vicentina, 09340 Ciudad de Mexico, DF, Mexico; ^3^Departamento de Ingenieria Electrica, Universidad Autonoma Metropolitana, Iztapalapa. Avenida San Rafael Atlixco 186, Colonia Vicentina, 09340 Ciudad de Mexico, DF, Mexico; ^4^Departamento de Biologia de la Reproduccion, Universidad Autonoma Metropolitana, Iztapalapa. Avenida San Rafael Atlixco 186, Colonia Vicentina, 09340 Ciudad de Mexico, DF, Mexico

## Abstract

The gastric mucosa ischemic tissular damage plays an important role in critical care patients' outcome, because it is the first damaged tissue by compensatory mechanism during shock. The aim of the study is to relate bioimpedance changes with tissular damage level generated by ischemia by means of confocal endomicroscopy and light microscopy. Bioimpedance of the gastric mucosa and confocal images were obtained from Wistar male rats during basal and ischemia conditions. They were anesthetized, and stain was applied (fluorescein and/or acriflavine). The impedance spectroscopy catheter was inserted and then confocal endomicroscopy probe. After basal measurements and biopsy, hepatic and gastric arteries clamping induced ischemia. Finally, pyloric antrum tissue was preserved in buffered formaldehyde (10%) for histology processing using light microscopy. Confocal images were equalized, binarized, and boundary defined, and infiltrations were quantified. Impedance and infiltrations increased with ischemia showing significant changes between basal and ischemia conditions (*P* < 0.01). Light microscopy analysis allows detection of general alterations in cellular and tissular integrity, confirming gastric reactance and confocal images quantification increments obtained during ischemia.

## 1. Introduction

Shock is a critical condition in which oxygen availability becomes restricted, and tissues will consume as much oxygen as is available. Compensated shock occurs when systemic delivery of oxygen (DO_2_) decreases and the tissues turn to anaerobic sources of energy. Under these conditions, cellular function is maintained as long as the combined yield of aerobic and anaerobic sources of energy provides sufficient ATP for protein synthesis and contractile processes. Some tissues are more resistant to hypoxia than others [1]. The intestinal and gastric mucosae show evidence of anaerobic metabolism before decreases in systemic oxygen consumption (VO_2_) is detected [2]. Uncompensated shock resulting in irreversible tissue damage occurs when the combined aerobic and anaerobic supplies of ATP are not sufficient to maintain cellular function. Failure of membrane-associated ion transport pumps, in particular those associated with the regulation of calcium and sodium, results in the loss of membrane integrity and in cellular swelling [3, 4]. Among other mechanisms that lead to irreversible cellular injury during hypoxia are depletion of cellular energy, cellular acidosis, and oxygen free radical generation [1]. 

An important objective in the care of critically ill patients is to ensure the adequacy of tissue oxygenation, since tissue hypoxia may result in anaerobic metabolism, cellular acidosis, and death [5]. Emerging data suggest that early aggressive resuscitation of critically ill patients may limit and/or reverse tissue hypoxia, progression to organ failure, and improve outcome [6]. In critical illness, blood flow to the splanchnic tissues is frequently reduced and redirected to other vital organs such as the brain, heart, and kidneys. Inadequately oxygenated, the splanchnic tissues may become prone to ischemia-related complications [7]. However, at present, there is no clinically useful method to directly monitor the level of gastric tissue dysoxia injury.

After a certain period of ischemia, the gastrointestinal tissue becomes vulnerable to damage due to reperfusion, in which restored oxygen supply produces free radical species, which lead to further tissue injury [8]. For an effective and correctly timed therapy, it may be very useful to know the ischemic level and the tissue damage in a continuous and simple manner [9].

Electric impedance measurements in tissues and biological systems have been used for decades in a wide variety of applications [10]. Impedance spectroscopy is the study of the passive electrical properties of biological tissues as a function of frequency. The impedance of biological tissues results from the interaction of an electrical current with the tissue at the cellular and molecular level. Impedance is the total effect of two separate orthogonal dimensions: the electrical resistance which restricts the flow of electrons and dissipates energy and the electrical reactance which is the capacity to store and release energy. 

On a conceptual level, the cell cytoplasm and extracellular space act as conductive media isolated from each other by the cell membrane. The conductivity of the extra- and intracellular space contributes to the overall resistance of the tissue while the cell membrane contributes to the capacitive effect [10, 11]. For instance, in a normoxic condition, a significant amount of low frequency current is able to flow through the extracellular spaces; when dysoxia occurs, the cells are not capable to generate enough energy to feed the ion pumps and extracellular water penetrates into the cell. As a consequence, the cells grow and invade the extracellular space causing a reduction of the current in extracellular fluids and are seen as a low frequency impedance increase [12]. Also the closure of gap junctions contributes to the impedance increment at these low frequencies. At high frequencies, impedance changes are influenced by intracellular and extracellular fluid impedances and ion permeability of cellular membranes [11, 13]. The electrical impedance of a living tissue can be continuously measured to determine its pathophysiological evolution. Some pathologies like ischemia, infarct, or necrosis cause cellular alterations that are reflected as impedance changes [14]. 

Our group developed an instrument that measures the impedance spectrum of the gastric mucosa in the range of 215 Hz to 1 MHz [15]. Along with the technique, an impedance spectrometry probe and nasogastric tube (ISP/NGT) [16] allow the direct acquisition of an electric impedance spectrum of the mucosa. Most of the authors in the bioimpedance field use the Cole-Cole equation to describe their experimental results characterizing the tissue bioimpedance with four parameters. For our device, an adaptation of Cole-Cole model was performed to develop an algorithm to calculate the 6 characteristic electrical values that best describe human gastric impedance measurements [17]. The device has been tested and validated during the last 12 years. The results confirm the potential of this technology for critical care monitoring. However, some studies are required to determine actual tissue damage levels via impedance spectroscopy in critical care patients in order to guide therapy and improve outcome. The objective of the study was to relate bioimpedance changes with tissular damage level generated during induced hypovolemic shock in a rat model, using confocal endomicroscopy and light microscopy.

## 2. Materials and Methods

### 2.1. Animals

Healthy adult male rats of Wistar strain, aged 3 months and weighting 350–400 g at the beginning of the experiment, were used. Animals were bred and kept at the Animal Facility of the Autonomous Metropolitan University in a temperature-controlled environment on a 12 : 12 h light-dark cycle and were fasted 12 h before experiment started. All procedures used in the study complied with the guide for care and use of laboratory animals. The experimental protocol was approved by the National Center for Medical Instrumentation and Imaging Research Ethical Committee.

### 2.2. Ischemia Induced Procedure

The surgical procedures were performed under clean conditions. Rats were deeply anesthetized using 1.5 mL intraperitoneal anesthesic cocktail (0.05 mL ketamine, 0.25 mL propionylpromazine, and 0.1 mL xylazine in 0.6 mL saline solution per mL). Another 1 mL of the ketamine/propionylpromazine/xylazine combination was kept for further maintenance anesthesia as needed.

**Figure 1:**
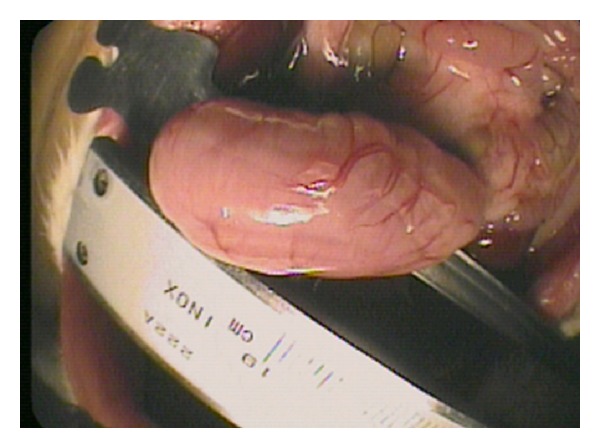
Stomach exhibition for greater curvature incision.

The animals were mounted on a dissection frame with attached limbs. After the onset of general anesthesia, 10 *μ*L/g of fluorescein (Alcon Pharma, Novartis Pharmaceuticals, Mexico) was injected in the tail vein. The rats underwent a 5 cm midline laparotomy from the xiphoid process to just above the penis. After opening the abdomen, the abdominal cavity was carefully inspected, and stomach was exposed (Figure 1). 

Using a 0.5 mL syringe containing heparin, blood samples were taken from the femoral artery immediately after opening the abdomen and 30 min after isquemia. pH and lactate concentration were assessed with a blood gas analyzer (GEM Premier 3000; Instrumentation Laboratory, Lexington, MA).

**Figure 2:**
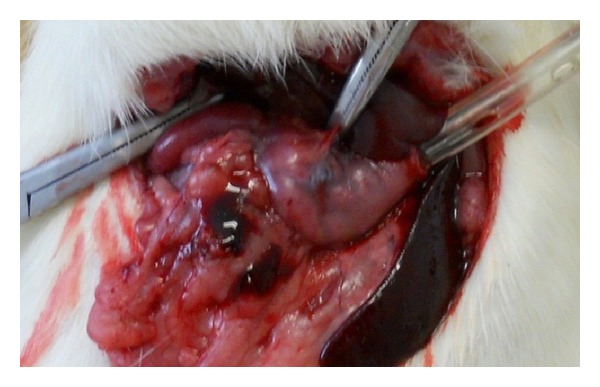
Hepatic and gastric arteries occlusion for ischemia generation.

Based on duodenum, a 1 cm incision was made over the greater curvature. Then a biopsy was obtained, and impedance measurements were taken during 15 minutes. When it was necessary, for topical staining, some drops of a 0.02% solution of acriflavine (Merck KGaA, Darmstadt, Germany) in saline were applied to the tissue surface, and excess dye was washed away with phosphate buffered saline. Then a collection of endomicroscopy images was stored. Afterwards, hepatic and gastric arteries were isolated and clamped (Figure 2). Bioimpedance measurements, confocal images, and biopsy were taken. After 30 min of ischemia, Evan's blue solution (1.5 mL of 0.1%) was injected into the celiac artery to evaluate perfusion. Once the experiment was concluded a humanitarian sacrifice was developed according to Mexican Official Standard (NOM-033-ZOO-1995).

### 2.3. Impedance Spectra Acquisition

The impedance spectroscopy system consists of three elements: the catheter, the impedance spectrometer, and the control and analysis system. The catheter is a flexible plastic tube that can be inserted in any hollow viscous organ. At the distal tip four electrodes are located that function as ionic current to electronic current transducers, such as Ag/AgCl electrodes. The electrodes are connected to leads that provide an electrical connection to the other end of the catheter along the wall of the tubing or in the lumen. At the proximal end, the leads end in an electrical multichannel connector that can be plugged into the impedance spectrometer [15]. The prototype impedance spectrometer meets the international regulation standards, as the BS EN 60601-1:1990 and ANSI/AAMI ES1:1993. A PC, with special software developed by our research group, controls the impedance spectrometer operations and data acquisition, storage and analysis. Impedance measurements obtained from these animals were automatically recorded every 30 seconds during 15 minutes for each condition.

### 2.4. Confocal Endomicroscopy Images Acquisition

Confocal endomicroscopy (EC-3870CIFK; Pentax, Tokyo, Japan) provides a 3-dimensional optical biopsy *in vivo* without physically disrupting epithelial integrity. For labeling of tissues fluorescent dyes (fluorescein and acriflavine) are used. Confocal gray scale images with a field of view of 475 × 475 *μ*m can be obtained by gently pressuring the confocal window onto the surface mucosa. The optical slice thickness of a single image is 7 *μ*m with a lateral resolution of 0.7 *μ*m. The range of the *z*-axis can be varied from the surface to 0–70 *μ*m below the surface layer of the gastric mucosa after topical staining with acriflavine. A single point within the tissue is scanned in a raster pattern, and measurement of light returning to the detector from successive points is digitized to construct an image of the scanned region. Each resultant image is a transverse optical section, 500 × 500 mm in size. Serial images are collected at a scan rate of 0.8 frames per second at a resolution of 1024 × 1024 pixels, approximating a 1000x magnification on a 19-inch screen. Five sets of confocal endomicroscopy images were obtained for each condition.

### 2.5. Tissue Collection and Histological Analysis

Chemical fixation of the pyloric antrum tissue for light microscopy was developed using buffered formaldehyde (10%) [18]. The pyloric antrum tissue was processed with the conventional histological techniques and included in Paraplast (Oxford Labware, St. Louis, Mo, USA). The pyloric antrum tissue was cut longitudinally and transversally; in each case, 5-micrometer serial sections were obtained and stained using hematoxylin-eosin [19]. The tissues sections were analyzed under a clear field light microscope (Axioskop II, Carl Zeiss) and image analyzer (Axiovision 4.8, Carl Zeiss). Thirty microscopic fields by condition were chosen at random. Micrographs were taken with an AxioCam MRc5 (Carl Zeiss).

### 2.6. Data Analysis

Impedance measurements were processed to calculate the central resistance and reactance at low frequencies (*R*_*L*_, and *X*_*L*_, resp.) and central resistance and reactance at high frequencies (*R*_*H*_ and *X*_*H*_ resp.), as the characteristic gastric impedance parameters of interest, as reported by Beltran et al. [17]. To analyze differences in impedance parameters between the different conditions (basal and ischemia), we calculated the average for each parameter over the monitoring time for each animal. A total of 50 to 100 images were collected per condition with the help of a foot pedal and digitally stored as gray-scale images. Analysis of images was performed using a specific algorithm designed by our group. Confocal endomicroscopy images were grouped by condition and staining type. Difference in the mean of all the values of experimental groups was analyzed using the Student's *t*-test. A *P* value < 0.01 was considered statistically significant. Data are presented as mean ± standard deviation (SD).

## 3. Results

The arteries clamping resulted in a significant decrease in blood pH (7.36 ± 0.04 for basal condition versus 7.18 ± 0.01 for ischemia condition). Lactate increased significantly to 5.23 ± 0.51 mmol/L, indicating metabolic acidosis and tissue ischemia.

In order to obtain impedance spectroscopy measurements and evaluate staining effects in spectra measurements, data were obtained using just acriflavine, just fluorescein, and the combination of fluorescein and acriflavine. Impedance parameters: *R*_*L*_ (central resistance at low frequencies), *X*_*L*_ (central reactance at low frequencies), *R*_*H*_ (central resistance at high frequencies), and *yX*_*H*_ (central reactance at high frequencies) were calculated, and Student's *t*-test was used in order to evaluate changes between basal and isquemia conditions.  Table 1 shows impedance parameters for acriflavine staining, Table 2 for fluorescein staining, and Table 3 for the combination of fluorescein and acriflavine. No immediate adverse reactions were noted following topical or systemic dye application.

**Figure 3:**
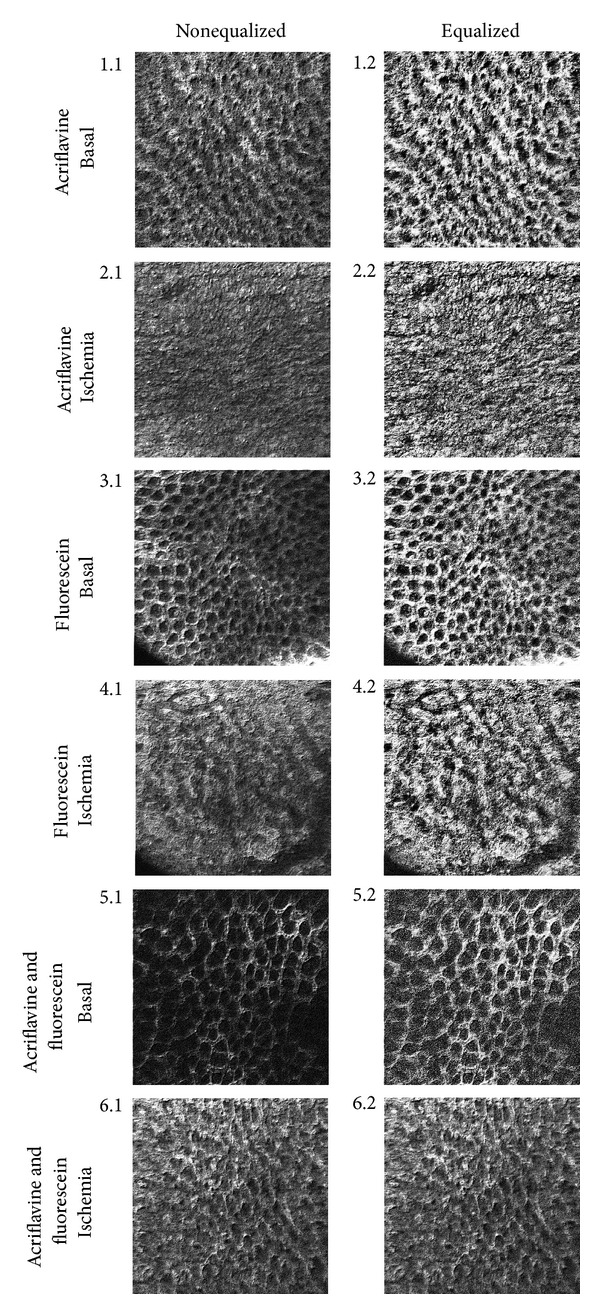
Confocal images comparison between nonequalized and equalized images, after edge enhancement for different staining.

**Figure 4:**
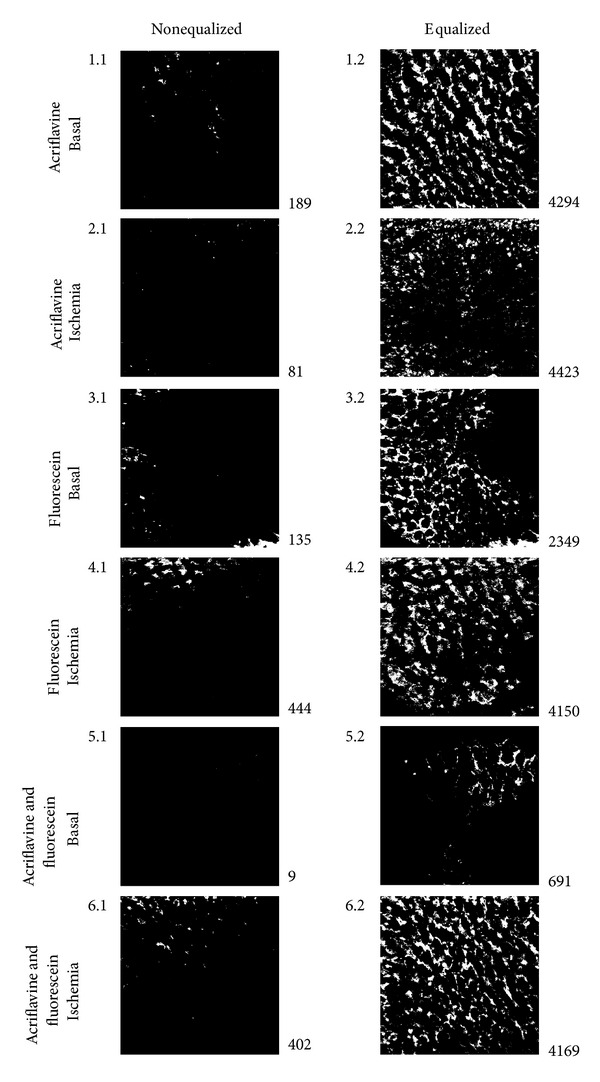
Number of imaging infiltrations according to staining type (right square), after binarization processing.

Confocal endomicroscopy images were grouped according to staining type, a set of 6 images was used for each staining, and 2 cut series (basal and ischemia) for the combination of fluorescein and acriflavine. Images were processed using an algorithm developed by our group. Equalization, filtering, and binarization processing were applied in order to enhance image features (Figure 3). The number of imaging infiltrations was calculated in Figure 4 (right corner), which indicate oedema alterations.  Table 4 illustrates the relationship between impedance parameters (*R*_*L*_ and *X*_*L*_) and number of infiltrations grouped by staining and condition. Impedance parameters increase during ischemia and are related to more infiltrations, especially for the combination of fluorescein and acriflavine staining.

### 3.1. Light Microscopy

**Figure 5:**
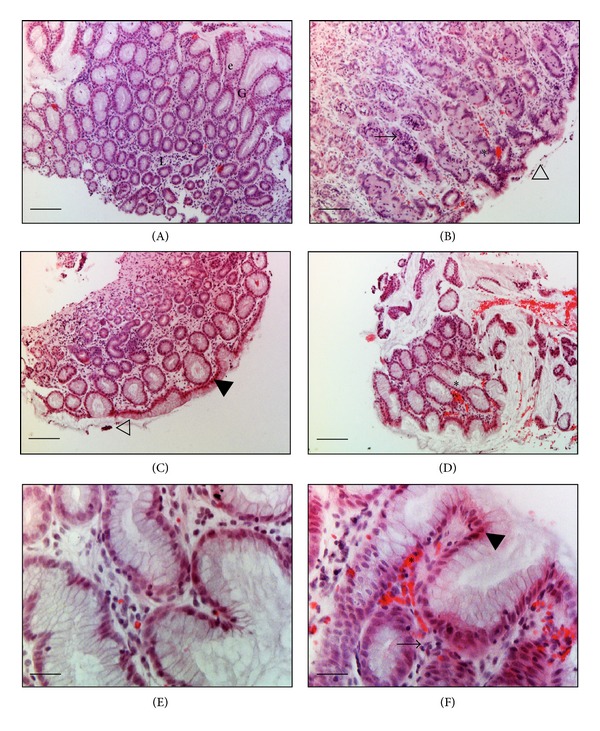
Hematoxylin and eosin stained histological sections of rat pyloric antrum tissue under basal and ischemia conditions. (A) and (E) basal condition: normal epithelium (e), lamina propria (L), and gastric glands (G). (B), (C), (D), and (F) ischemia condition: some epithelial cells show histological features of necrosis (▲), and the initial phases of the acute inflammatory response: leukocyte infiltration (→), vascular congestion (∗), and epithelium integrity lost (*∇*) ((A), (B), (C), and (D) magnification ×100, Bar = 100 *μ*m; H-E) ((E) and (F) magnification ×400, bar = 25 *μ*m).

In basal condition the gastric mucosa presents simple columnar epithelium with invaginations to form foveolae and glands. A thin lamina propria layer forms packing among many gastric glands (Figures 5(A) and 5(E)). 

In ischemic animals evidence of sub-lethal (pale stained) and lethal cell injury (cytoplasmic eosinophilia, and nuclear condensation) are observed in Figures 5(C) and 5(F) respectively. The lethal damage generates cellular death. Few scattered mononuclear cells (neutrophils), mainly localized between the crypts and diffusely distributed through the lamina propria, and vessels dilatation are observed (Figures 5(B), 5(C), and 5(D)). These are events during the initial phases of the acute inflammatory response. Also slightly foveolar epithelium erosion is illustrated in Figures 5(B) and 5(C).

## 4. Discussion and Conclusion

Up to now, no clinically useful method to directly monitor the level of tissue dysoxia injury is available. We proposed that bioimpedance and particularly gastric reactance is an early indicator of ischemia and we developed some studies that confirm the prognostic and diagnostic value of these measurements [20, 21]. However, before we may advocate the clinical use of this technology we need to first answer the following questions: (1) Can we quantify oedema and tissue damage from impedance parameters?, (2) Can we use this information to improve patient management and treatment? The second question is the hardest to answer and will require further research and experience. This study was designed to answer the first question and relate impedance parameters changes with tissular damage level generated during induced ischemia in a rat model, using confocal endomicroscopy and light microscopy.

This is the first animal model designed to quantify gastric injury generated by ischemia by confocal endomicroscopy imaging and evaluate its relationship with bioimpedance measurements. In this hypovolemic shock model impedance parameters statistically increase during ischemia as Tables 1, 2, and 3 showed. The greater changes are observed in central resistance at low frequencies that reflects tissue oedema caused by prolonged ischemia, causing a net increase in intracellular to extracellular volume ratio. Accumulation of metabolic products, cell swelling caused by osmosis, and closing gap junctions are important effects which can be detected by means of electrical impedance spectroscopy: at low frequencies (<1 kHz) tissue impedance is influenced mostly by extracellular fluid impedance (extracellular pathway narrowing (caused by cell swelling) and gap junction status can be ascertained), while at high frequencies it is influenced by both intercellular and extracellular fluid impedances [11, 13]. According to confocal endomicroscopy imaging processing we developed an algorithm to quantify indirectly cell swelling by infiltration accumulation. Although acriflavine has been used to highlight nuclei, when it is used alone, no differences in infiltrations were found between basal and ischemia conditions (Table 4). Nuclear staining was diminished in necrotic areas generated during ischemia. Because the inflammatory process was focal rather than homogeneous, *in vivo* microscopy helped to localize the inflammatory infiltrate and necrotic areas. The inflammatory infiltration and weak nuclear pyloric antrum tissue staining of the foci corresponded well with *ex vivo* histology using hematoxylin and eosin staining as reported in other confocal endomicroscopy studies [22].

Fluorescein staining exhibits significant differences (*P* < 0.01) between basal and ischemic groups; however there were a large number of infiltrations for basal conditions that are not related with the normal pyloric antrum tissue evidenced by histology analysis (Figure 5). The staining that best reflects ischemia condition and that can be quantified properly by our algorithm is the combination of fluorescein and acriflavine. In this case, our algorithm made a clear differentiation between normal and inflammatory infiltrations in pyloric antrum tissue as observed in Table 4. Relatively few studies have therefore successfully used this technique for imaging in animal disease models or in humans *in vivo* [22]. On the other hand, targeted fluorescence technology has been thoroughly exploited but has not been linked to *in vivo* microscopic imaging [23].

Impedance parameters increased during ischemia; however there is not a clear effect of staining type in these measurements. It seems like fluorescein alone lightly decreases *R*_*L*_, but we did not find a clear relationship between impedance parameters and inflamatory infiltration numbers. Nevertheless, there was a relation between impedance increase and cellular damage observed by light microscopy (Figure 5), indicating that the impedance spectrometer device proposed will be useful to reflect tissue oedema in critically ill patients. Further studies are necessary in order to understand cellular function and interactions in human diseases to improve patient management and treatment. The results demonstrated that the infiltration number calculated is associated with the tissue injury observed histologically. This method could be a quick evaluation for the tissue status that realizes the concept of electrical biopsy [24].

## Figures and Tables

**Table 1:** Impedance parameters for acriflavine staining. Difference in the mean parameters by condition was analyzed using the Student's *t*-test. Data are presented as mean ± standard deviation (SD).

Impedance parameter	Basal	Ischemia	*P*
*R*_*L*_ [Ohms]	75.4 ± 1.7	91.9 ± 1.4	<0.01*
*R*_*H*_ [Ohms]	34.3 ± 0.7	35.9 ± 0.5	0.053
*X*_*L*_ [-jOhms]	7.4 ± 0.5	11.5 ± 0.4	<0.01*
*X*_*H*_ [-jOhms]	8.9 ± 0.2	18.5 ± 0.1	0.037

*Statistically significant (*P* < 0.01).

**Table 2:** Impedance parameters for fluorescein staining. Difference in the mean parameters by condition was analyzed using the Student's *t*-test. Data are presented as mean ± standard deviation (SD).

Impedance parameter	Basal	Ischemia	*P*
*R*_*L*_ [Ohms]	62.9 ± 3.1	99.1 ± 3.4	<0.01*
*R*_*H*_ [Ohms]	31.5 ± 1.5	39.2 ± 1.6	<0.01*
*X*_*L*_ [-jOhms]	3.1 ± 0.3	6.7 ± 0.4	<0.01*
*X*_*H*_ [-jOhms]	10.9 ± 0.8	17.5 ± 0.8	<0.01*

*Statistically significant (*P* < 0.01).

**Table 3:** Impedance parameters for the combination of fluorescein and acriflavine. Difference in the mean parameters by condition was analyzed using the Student's *t*-test. Data are presented as mean ± standard deviation (SD).

Impedance parameter	Basal	Ischemia	*P*
*R*_*L*_ [Ohms]	70.3 ± 2.1	91.3 ± 1.6	<0.01*
*R*_*H*_ [Ohms]	34.7 ± 0.6	35.6 ± 0.5	0.278
*X*_*L*_ [-jOhms]	7.6 ± 0.4	11.7 ± 0.3	<0.01*
*X*_*H*_ [-jOhms]	9.4 ± 0.4	16.3 ± 0.3	<0.01*

*Statistically significant (*P* < 0.01).

**Table 4:** Relationship between impedance parameters and confocal images infiltration numbers. Data are presented as mean ± standard deviation (SD).

	*R*_*L*_ [Ohms]	*X*_*L*_ [-jOhms]	Infiltration number
Acriflavine			
Basal	75.4 ± 1.7	7.4 ± 0.5	4294
Ischemia	91.9 ± 1.4	11.5 ± 0.4	4423
Fluorescein			
Basal	62.9 ± 3.1	3.1 ± 0.3	2349
Ischemia	99.1 ± 3.4	6.7 ± 0.4	4150
Acriflavine and fluorescein			
Basal	70.3 ± 2.1	7.6 ± 0.4	691
Ischemia	91.3 ± 1.6	11.7 ± 0.3	4169
